# Eliminating Absence Seizures through the Deep Brain Stimulation to Thalamus Reticular Nucleus

**DOI:** 10.3389/fncom.2017.00022

**Published:** 2017-04-19

**Authors:** Zhihui Wang, Qingyun Wang

**Affiliations:** Department of Dynamics and Control, Beihang UniversityBeijing, China

**Keywords:** absence seizures, spike and slow-wave discharges, mean-field model, deep brain stimulation, dynamical transition

## Abstract

Deep brain stimulation (DBS) can play a crucial role in the modulation of absence seizures, yet relevant biophysical mechanisms are not completely established. In this paper, on the basis of a biophysical mean-field model, we investigate a typical absence epilepsy activity by introducing slow kinetics of GABA_B_ receptors on thalamus reticular nucleus (TRN). We find that the region of spike and slow-wave discharges (SWDs) can be reduced greatly when we add the DBS to TRN. Furthermore, we systematically explore how the corresponding stimulation parameters including frequency, amplitude and positive input duration suppress the SWDs under certain conditions. It is shown that the SWDs can be controlled as key stimulation parameters are suitably chosen. The results in this paper can be helpful for researchers to understand the thalamus stimulation in treating epilepsy patients, and provide theoretical basis for future experimental and clinical studies.

## 1. Introduction

Absence epilepsy, a generalized non-convulsive epilepsy, can be observed from the electroencephalogram (EEG). A sudden, brief loss of consciousness with bilaterally synchronous 2–4 Hz SWDs is the main feature of absence seizures, which mainly affects children and young adolescents (Crunelli and Leresche, 2002). Panayiotopoulos (2008) found that absence seizures propagated across the whole cortex and happened without any prediction. The possible involvement of the thalamus during epileptic seizures has been shown in the human recordings (Jasper and Kershman, 1941; Prevett et al., 1995). After that, a series of electrophysiological recordings in experimental models showed the complexity of their dynamical characteristics and corresponding biophysics mechanisms of so-called corticothalamic system (Avoli and Gloor, 1981; Steriade et al., 1993a,b; Seidenbecher et al., 1998). A large number of results on the generalized non-convulsive epilepsy have shown that the generation of SWDs is due to the abnormal interactions between the cerebral cortex and thalamus (Marescaux and Vergnes, 1995; Timofeev and Steriade, 2004). And some studies of computational modeling also have been utilized to investigate more deep insights into the possible generation mechanisms of SWDs in the corticothalamic system (Breakspear et al., 2006; Marten et al., 2009; Chen et al., 2014).

Mean-field modeling can be useful to study the dynamical behaviors of collective neurons. It has been increasingly used to investigate the evolution behaviors of epileptic seizures, especially for absence seizures. For examples, Robinson et al. (2002) focused on understanding the transition from the steady state to the typical SWD state by means of the constructed mean-field model. In addition to the classical SWD activities, Marten et al. (2009) demonstrated much more dynamical phenomena including poly-spike and wave, wave spike or even no discernible spike-wave onset in the recorded EEG data. Furthermore, Destexhe (1998) suggested that the SWDs could arise from thalamocortical loops in which the corticothalamic feedback indirectly evoked GABA_B_-mediated inhibition in the thalamus. Based on the dynamical bifurcations of the mean-field model in the brain, Breakspear et al. (2006) explained critical features of the generalized epilepsies. Recently, Chen et al. (2014) discovered a computational evidence for the bilateral control of absence seizures by simulating the typical SWD morphology, which once again highlights the critical role of mean-field model.

There are many different methods in the treatment of epilepsy, and pharmacotherapy is currently the primary modality (Löscher, 2002). Another techniques such as surgical resection of epileptogenic foci and neuromodulatory techniques have also been used. Especially for the case of intractable epilepsy, surgical intervention has been developed rapidly because of technological advancement. In general, 86 percent of patients were satisfied with their postoperative results, which were supported by previous statistical work (Macrodimitris et al., 2011). Even when the patients suffered from the seizures again after the surgery, 90 percent of patients stated that the surgical treatment was the right decision (Reid et al., 2004). Although surgical intervention can be well accepted by epilepsy patients, complications of surgery are still the potential risks. By contrast, DBS sends electrical pulses to the implanted electrodes that are placed in specific areas of the brain to control seizures. This method was found to be effective to modulate the epileptic brain activity in some animal experiments, especially acting on the thalamus (Mina et al., 2013). Nanobashvili et al. (2003) showed that the stimulation of thalamic reticular nucleus (TRN) suppressed the development of seizures that was taken from Male Wistar rats. In addition, many similar studies have indicated that the high-frequency stimulation of TRN could obtain an anti-epileptogenic effect and interrupt abnormal electroencephalogram recordings in rats (Pantoja-Jiménez et al., 2014). Hence, the above animal experiments suggest a new approach for seizure control in treating epilepsy. DBS might become a very attractive and meaningful method to treat epilepsy patients, because it is relative safe and reversible (Huang and van Luijtelaar, 2016). Thus, compared with the other neurosurgical treatments, DBS needs the complicated implantation procedures and advanced techniques to localize the suitable target in the brain (Fuentes et al., 2009; Lega et al., 2010).

According to the above findings, we develop a mean-field model for the epileptic absence seizures through adding the DBS to TRN, and analyze its neurophysiological effects on epileptic cortical dynamics. By means of dynamical analysis, we explore how the slow kinetics of GABA_B_ receptors on TRN induce the typical absence epilepsy activities. Furthermore, we also study how the typical SWD oscillation activities are modulated and terminated via changing the range of relevant stimulation parameters (i.e., frequency, amplitude and positive input duration). These results in this paper can further highlight the critical roles of thalamus stimulation in eliminating absence seizures, and can provide useful therapeutic method for the brain disease.

## 2. Description of models

**Figure 1:**
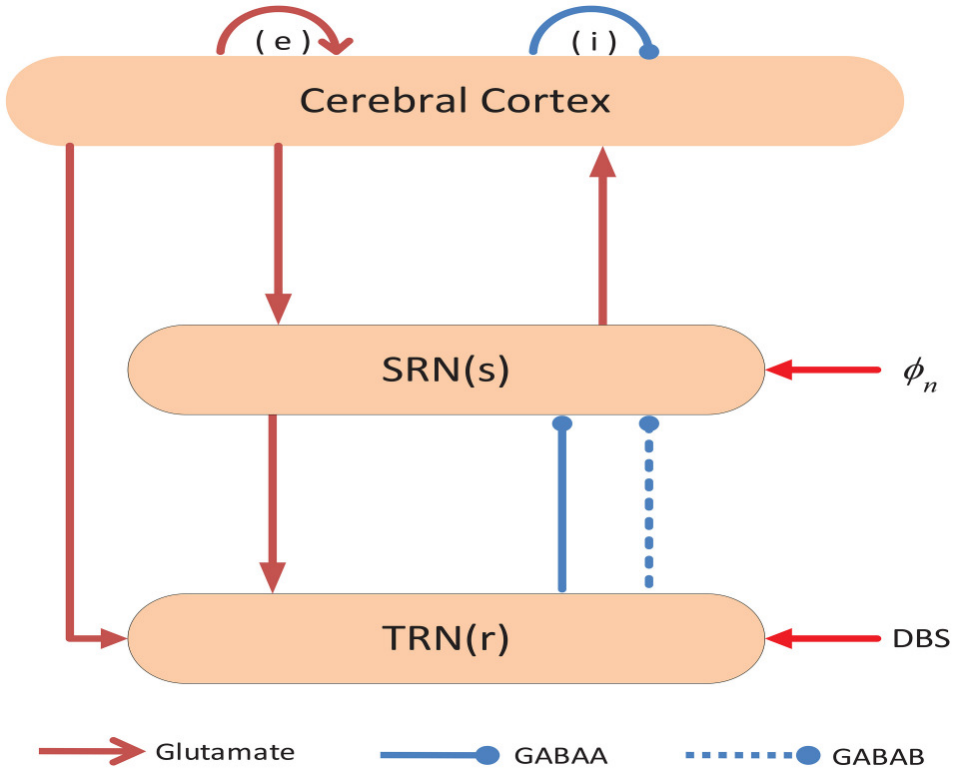
**Framework of the cortico-thalamic network**. The network contains two components: (I) the cerebral cortex, (II) the thalamus. Neural populations include: e = excitatory pyramidal neurons (EPN); i = inhibitory interneurons (IIN); r = thalamus reticular nucleus (TRN); s = specific relay nuclei (SRN). Excitatory projections are mediated by glutamate, which are denoted by the red lines with arrow heads, while the inhibitory projections are mediated by GABA_A_ and GABA_B_ receptors, which are represented by blue solid and dashed lines with round heads, respectively.

In order to simulate the EEG activity during absence seizures, we build a model to describe the population dynamics in two distinct brain regions, namely the thalamus and the cerebral cortex. The network connection schematic of this model has been presented in Figure 1, which includes four neural populations: e = excitatory pyramidal neurons (EPN); i = inhibitory interneurons (IIN); r = thalamus reticular nucleus (TRN); s = specific relay nuclei (SRN) (Marten et al., 2009; Chen et al., 2014). There are three types of neural projections in the network. For the sake of simplicity, we distinguish them in different types of lines and heads. Excitatory projections are mediated by glutamate, which are denoted by the red lines with arrow heads, while inhibitory projections are mediated by GABA_A_ and GABA_B_ receptors, which are represented by the blue solid and dashed lines with round heads, respectively. Additionally, DBS denotes the deep brain stimulation, which is a neuromodulator technique.

Based on the important roles of cortex and thalamus during epileptic seizures (Marescaux and Vergnes, 1995; Timofeev and Steriade, 2004; Breakspear et al., 2006), we modify a mean-field model (Marten et al., 2009; van Albada and Robinson, 2009; Chen et al., 2014) by removing the basal ganglia and adding the DBS to TRN to explore the macroscopic dynamics of neural populations. For each neural population, the mean firing rate *Q*_*a*_ is described by an increasing sigmoid function of the mean membrane potential *V*_*a*_, and given by (Robinson et al., 1997, 1998),

(1) $$Q_{a}(r,t)=F[V_{a}(r,t)]=\frac{Q_{a}^{max}}{1+exp[-\frac{\pi }{\sqrt{3}}\frac{\left(V_{a}(r,t)-\theta_{a}\right)}{\sigma }]},$$

where *a* ∈ *A* = {*e, i, r, s*} refer to different neural populations, $Q_{a}^{max}$ represents the maximum firing rate, θ_*a*_ is the mean threshold potential, σ is the standard deviation of firing thresholds, and **r** denotes the spatial position. With an average firing rate *Q*_*a*_, the neural population fires action potentials when *V*_*a*_ exceeds the threshold θ_*a*_. As the sigmoid shape of *Q*_*a*_ is physiologically crucial for the model, the average firing rate cannot exceed the maximum firing rate $Q_{a}^{max}$. The change of the average membrane potential for neurons in type *a* can be modeled as van Albada et al. (2009),

(2) $$D_{\alpha \beta }V_{a}(r,t)=\sum_{b\in A}v_{ab}\cdot \phi_{b}(r,t),$$

(3) $$D_{\alpha \beta }=\frac{1}{\alpha \beta }[\frac{\partial^{2}}{\partial t^{2}}+(\alpha +\beta )\frac{\partial }{\partial t}+\alpha \beta ],$$

where the differential operator *D*_αβ_ represents the synaptic and dendritic filtering of incoming signals, α and β denote the decay and rise rate of the cell-body potential, respectively (van Albada et al., 2009). ϕ_*b*_(**r**, *t*) is the incoming pulse rate and *v*_*ab*_ is connection strength from the neural population of type b to type a. For the sake of simplicity, we ignore the transmission delay among some neural populations including the delay between cortex and thalamus in our model. Nevertheless, some animal experiments have shown that the slow kinetics of inhibitory GABA_B_ receptors on TRN can result in the generation of absence seizures (Destexhe, 1998; Chen et al., 2014). Hence, to investigate the effect of slow kinetics of inhibitory GABA_B_ receptors on absence seizures, we introduce two connections ϕ_*b*_(**r**, *t*) and ϕ_*b*_(**r**, *t*−τ) from TRN to SRN, which are used to denote the inhibitory GABA_A_ and GABA_B_ synapses. The postsynaptic currents of GABA_B_ functions develop on a slower time scales than the currents of GABA_A_ since the GABA_B_ functions via second messenger processes. Therefore, the GABA_B_-mediated inhibitory projection will be delayed with respect to GABA_A_ when the thalamic neurons fire.

In the cerebral cortex, the propagation of cortical excitatory axonal field ϕ_*e*_ can be well-approximated by a damped wave equation (Jirsa and Haken, 1996),

(4) $$\frac{1}{\gamma_{e}^{2}}[\frac{\partial^{2}}{\partial t^{2}}+2\gamma_{e}\frac{\partial }{\partial t}+\gamma_{e}^{2}-v_{e}^{2}\nabla^{2}]\phi_{e}(r,t)=Q_{e}(r,t),$$

where ∇^2^ is the Laplacian operator and *Q*_*e*_ is the excitatory firing rates. $\gamma_{e}=\frac{v_{e}}{r_{e}}$ is the temporal damping rate of pulses, consisting of the characteristic range *r*_*e*_ of axons and the mean conduction velocity *v*_*e*_ of excitatory neurons. Except for the axons of cortical excitatory pyramidal neurons, other axons are too short to provide wave propagation on the relevant scales. Here, ϕ_*c*_ = *F*(*V*_*c*_)(*c* = *i, r, s*). Moreover, the absence seizures, typical generalized seizures, the dynamical activities are regarded as global brain activities. Thus a reasonable hypothesis can be proposed that the spatial activities are unified. For this purpose, the spatial derivative can be ignored and then we can set ∇^2^ = 0 in Equation (4). Accordingly, the propagation effect of ϕ_*e*_ can be finally given by,

(5) $$\frac{1}{\gamma_{e}^{2}}[\frac{\partial^{2}}{\partial t^{2}}+2\gamma_{e}\frac{\partial }{\partial t}+\gamma_{e}^{2}]\phi_{e}(r,t)=Q_{e}(r,t).$$

For cortical neuronal populations, We assume that intracortical connectivities are proportional to the numbers of synapses involved (Robinson et al., 2002; van Albada et al., 2009), which implies *V*_*i*_ = *V*_*e*_, and thus *Q*_*a*_ = *F*(*V*_*a*_) implies *Q*_*i*_ = *Q*_*e*_. Although the model has been simplified in many aspects, it doesn't affect the results of this work.

In the end, we will introduce the DBS, which can be described as a periodic step function with the following form (Fan et al., 2016),

(6) $$DBS(t)=a\times H(sin(\frac{2\pi t}{\rho }))(1-H(\frac{sin(2\pi (t+\delta ))}{\rho })).$$

**Figure 2:**
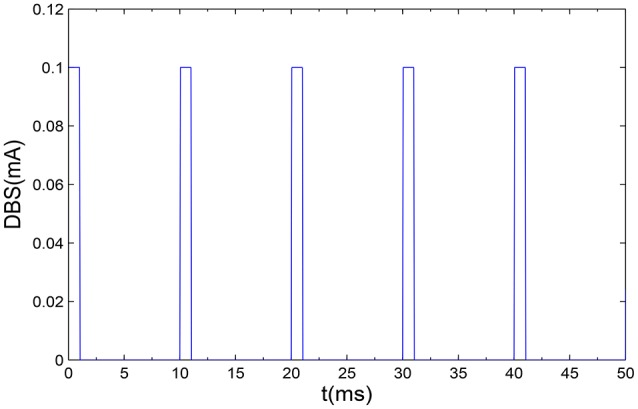
**A illustrative example of monophasic current pulses DBS are applied to the proposed model**. Here, we set the amplitude *a* = 0.1 mA, the frequency *f* = 100 Hz, and the positive input duration δ = 1 ms.

Where *H* is the Heaviside bi-value step function, which satisfies that *H*(*x*) = 1 if *x* > 0 and *H*(*x*) = 0 if *x* ≤ 0. *a* and ρ is the amplitude and period, respectively. δ is the duration of positive input which determines duty circle of positive pulse. In order to simulate the non-regular property of DBS, the instantaneous frequency *f* of incoming pulses can be expressed as $\frac{1}{T}$ according to the previous literature (So et al., 2012). A series of monophasic current pulses have been shown as in Figure 2.

When the DBS is applied on the TRN, we will rewrite above Equations (1–6) in the first-order form for all neural populations. The final mathematical description of the model is given as follows,

(7) $$\frac{d\phi_{e}(t)}{dt}={\dot{\phi }}_{e}(t),$$

(8) $$\frac{d{\dot{\phi }}_{e}(t)}{dt}={\gamma_{e}}^{2}[-\phi_{e}(t)+F(V_{e}(t))]-2\gamma_{e}{\dot{\phi }}_{e}(t),$$

(9) $$\frac{dV_{e}(t)}{dt}={\dot{V}}_{e}(t),$$

(10) $$\begin{array}{l} \frac{d{\dot{V}}_{e}(t)}{dt}=\alpha \beta [v_{ee}\phi_{e}(t)+v_{ei}F(V_{i}(t))+v_{es}F(V_{s}(t))-V_{e}(t)]\\ \;-\;(\alpha +\beta ){\dot{V}}_{e}(t), \end{array}$$

(11) $$\frac{dV_{r}(t)}{dt}={\dot{V}}_{r}(t),$$

(12) $$\begin{array}{l} \frac{d{\dot{V}}_{r}(t)}{dt}=\alpha \beta [v_{re}\phi_{e}(t)+v_{rs}F(V_{s}(t))-V_{r}(t)]-\;(\alpha +\beta ){\dot{V}}_{r}(t)\\ \;+\;DBS(t), \end{array}$$

(13) $$\frac{dV_{s}(t)}{dt}={\dot{V}}_{s}(t),$$

(14) $$\begin{array}{l} \frac{d{\dot{V}}_{s}(t)}{dt}=\alpha \beta [\begin{array}{l} v_{se}\phi_{e}(t)+v_{sr}^{A}F(V_{r}(t))+v_{sr}^{B}F(V_{r}(t-\tau ))\\ \;-\;(V_{s}(t)-\phi_{n}) \end{array}]\\ \;-\;(\alpha +\beta ){\dot{V}}_{s}(t). \end{array}$$

Here ϕ_*n*_ is the constant nonspecific subthalamic input onto SRN, τ is GABA_B_ delay from TRN to SRN.

Unless otherwise mentioned, the values of parameters we used have been shown in Table 1. The first nine different parameters are approximately estimated from physiological experiments, and they have been applied in some literatures (Rennie et al., 2000; Robinson et al., 2001; Zhao and Robinson, 2015). The range of delay τ is set as [0, 180] ms, which is estimated from a number of different subjects with absence epilepsy (Marten et al., 2009). However *v*_*ab*_ (*a, b* ∈ *A* = {*e, i, r, s*}) are less well known from physiology (Robinson et al., 2002), which leads to a quite wide range of *v*_*ab*_. In addition, we will change some critical parameters within certain ranges (i.e., *v*_*sr*_, τ, *f*, α, and δ) to acquire different dynamical features and explore the possible effects of the parameters on controlling absence seizures.

**Table 1:** **Model parameters**.

**Symbol**	**Description**	**Value**
$Q_{e}^{max}$, $Q_{i}^{max}$	Cortical maximum firing rate	250 Hz
$Q_{r}^{max}$	TRN maximum firing rate	250 Hz
θ_*e*_, θ_*i*_	Mean firing threshold of cortical populations	15 mV
θ_*s*_	Mean firing threshold of SRN	15 mV
θ_*r*_	Mean firing threshold of TRN	15 mV
γ_*e*_	Cortical damping rate	100 Hz
α	Synaptodendritic decay time constant	50 *s*^−1^
β	Synaptodendritic rise time constant	200 *s*^−1^
σ	Threshold variability of firing rate	6 mV
τ	Time delay due to slow synaptic kinetics of GABA_B_	50 ms
ϕ_*n*_	Nonspecific subthalamic input onto SRN	2 mVs
*v*_*ee*_	Self-coupling strength of EPN	1 mVs
−*v*_*ei*_	Coupling strength from IIN to EPN	1.8 mVs
*v*_*re*_	Coupling strength from EPN to TRN	0.05 mVs
*v*_*rs*_	Coupling strength from SRN to TRN	0.5 mVs
$-v_{sr}^{A,B}$	Coupling strength from TRN to SRN	0.4–2 mVs
*v*_*es*_	Coupling strength from SRN to EPN	1.8 mVs
*v*_*se*_	Coupling strength from EPN to SRN	2.2 mVs

All of the numerical simulations are performed in the MATLAB (MathWorks, USA) environment. Similar to the previous works (Chen et al., 2014, 2015), the delay differential equations in the model are solved by the standard fourth-order Runge-Kutta method. The fixed temporal resolution of numerical integration is 0.05 ms. All the simulations are performed up to 15 s and the data from 5 to 15 s are used for statistical analysis. We don't consider the effect of noise. What's more, the step length of integrate is small enough to ensure the numerical accuracy and reliability in the simulations.

## 3. Results

### 3.1. Generation of the typical absence epilepsy activities by introducing pathological mechanisms

**Figure 3:**
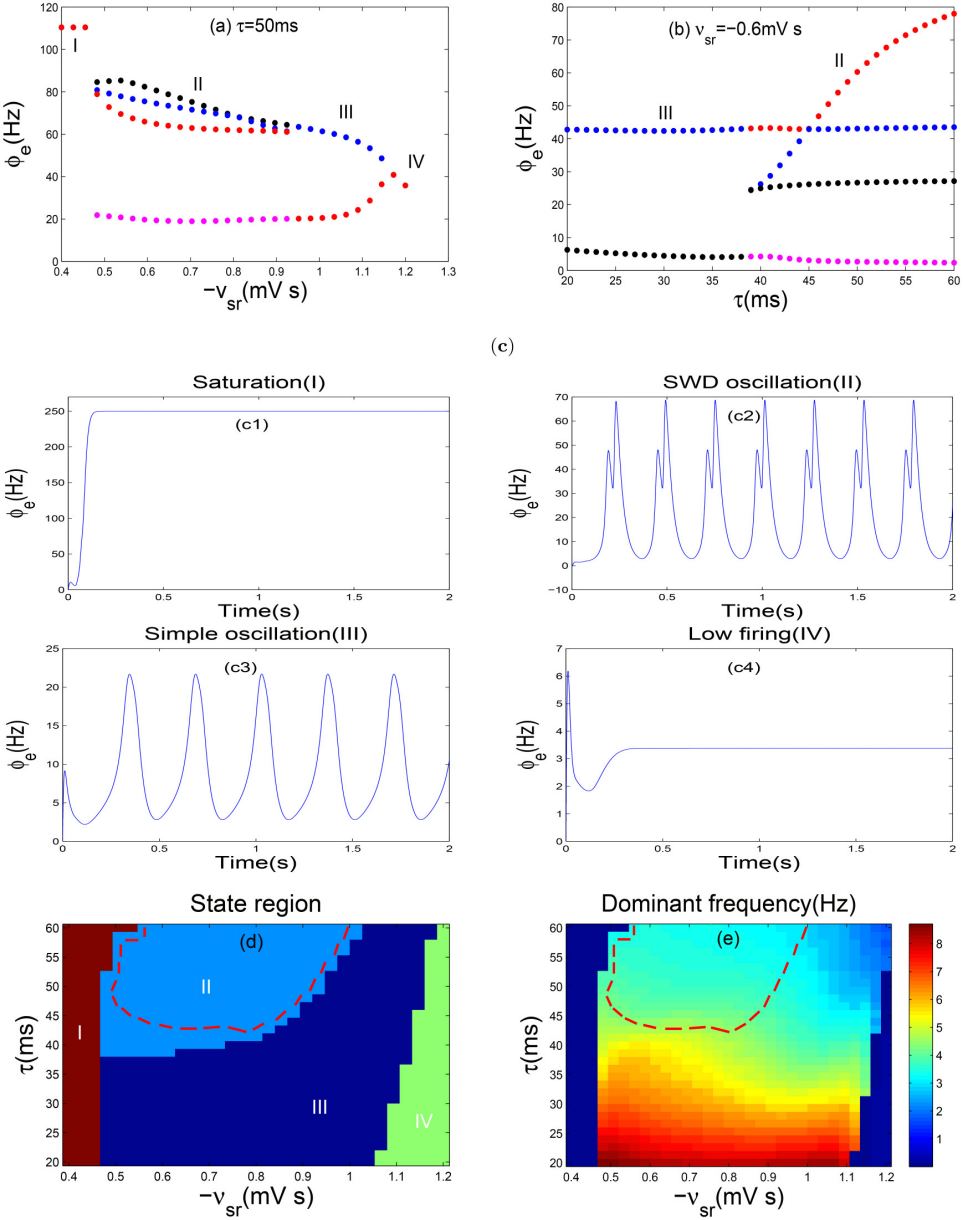
**Absence seizures induced by coupling strength of TRN-SRN pathway and the slow dynamics of GABA_B_ receptorS in TRN**. **(a,b)** The bifurcation diagrams in ϕ_*e*_, are obtained by varying −*v*_*sr*_ **(a)** and τ **(b)**. Here −*v*_*sr*_ represents the inhibitory coupling strength of TRN-SRN pathway, whereas τ denotes the GABA_B_ delay. In order to observe the bifurcation phenomenon obviously, the ordinate scale is used exactly in a log scale in **(a)**. Four types of dynamics state regions, including the saturation region (I), the SWD oscillation region (II), the simple oscillation region (III), the low firing region (IV) are indicated. **(c)** The typical time series of ϕ_*e*_ are corresponding to the above four different dynamics states. Here we choose −*v*_*sr*_ = −0.4 mV s **(c1)**, −*v*_*sr*_ = −0.6 mV s **(c2)**, −*v*_*sr*_ = −1.1 mV s **(c3)**, −*v*_*sr*_ = −1.2 mV s **(c4)** and set τ = 50 ms. **(d,e)** Two-dimension bifurcation diagram **(d)** and dominant frequency diagram **(e)** in the (−*v*_*sr*_, τ) panel. Different colors in **(d)** represent the different dynamical states. The region surrounded by red dashed lines in **(d)** represents the SWD oscillation regions, which falls into the 2–4 Hz frequency range in **(e)**.

Recent studies have reported that the slow kinetics of GABA_B_ receptors on TRN is an important pathological factor in destroying the normal oscillatory patterns of the corticothalamic system (Hosford et al., 1992; Destexhe, 1998; Timofeev and Steriade, 2004). Inspired by these findings, we will explore the impact of the slow kinetics of GABA_B_ receptors on epileptic seizures by means of the reasonable model as given above. Initially, we plot one-dimensional bifurcation diagrams as the inhibitory coupling strength −*v*_*sr*_ and the GABA_B_ delay τ are changed, respectively (see Figures 3a,b). In particular, the bifurcation diagrams are obtained by plotting the stable local minimum and maximum values of cortical excitatory axonal fields ϕ_*e*_. As shown in Figure 3a, there are four different dynamical states including the saturation region (I), the SWD oscillation region (II), the simple oscillation region (III) and the low firing region (IV) as −*v*_*sr*_ is changed. For more clear vision, some typical time series are depicted in Figures 3c1–c4 with some fixed −*v*_*sr*_, respectively, which support the results of Figure 3a. It should be noted that the saturation state is a non-physiological brain state, whose threshold is set as 250 Hz in our model. In addition, when the coupling strength −*v*_*sr*_ is too strong, the TRN almost inhibits the firing of SRN. In this case, the model transfers into the low firing states and no oscillation phenomenon can be found. With decreasing the coupling strength −*v*_*sr*_, we find an approximate 3 Hz simple oscillation at −*v*_*sr*_ ≈ 1.16*mVs*, which is similar to oscillatory EEG signals at the onset of absence seizures in some patients (Rodrigues et al., 2005). And their modes just contain approximately 3 Hz sine-like oscillations and don't include any spikes. Hence, the simple oscillation actually may lead to a pathological system. And the state transition between the simple and SWD oscillation has been shown in Figure 3a. When the coupling strength −*v*_*sr*_ is too weak, the inhibition from TRN hardly suppresses the firing of SRN. The strong excitatory from cortical pyramidal neurons makes firing of the SRN arrive a high level immediately. The high excitatory level promotes the firing of cortical neurons, and reaches the saturation states (region I). From Figure 3b, we can observe two types of oscillation patterns as the delay τ is changed: the SWD oscillation (region II) with multiple pairs of maximums and minimums and the simple oscillation (region III) with only one pair of maximums and minimums at the fixed delay parameter.

Furthermore, as shown in Figure 3d, four different state regions are displayed in two-parameter space (−*v*_*sr*_, τ), and the SWD oscillation state is represented by region II. The dominant frequency is estimated using the power spectral analysis, which is obtained from the time series of the excitatory axonal fields ϕ_*e*_ via fast Fourier transform. Meanwhile, the maximum peak frequency is defined as the dominant frequency of neural oscillation. In particular, Figure 3e shows the frequency range of epileptic seizures within 2–4 Hz. It should be noted that the region surrounded by the red dashed lines in Figure 3d represents the 2–4 Hz SWD oscillation region, i.e., absence seizures region (pathological region). We also find that the system can generate the 2–4 Hz SWD activities when the range of −*v*_*sr*_ belongs to (0.47, 1.04)*mVs* and the parameter τ is larger than 40 ms as shown in Figure 3d. Hence, when the system is located in the region II within the red line, the decrease of τ can lead a transition from the SWD to simple oscillation state. However, both decreasing and increasing the inhibitory coupling strength −*v*_*sr*_ can also suppress the SWDs. In addition, increasing the parameters −*v*_*sr*_ and τ simultaneously can decrease the dominant frequency of neural oscillations only for the simple and SWD oscillation states (Figure 3e). Therefore, we obtain that the GABA_B_ delay might has a greater impact on the dominant frequency than the coupling strength −*v*_*sr*_. So, a relatively long delay is required to generate the SWD oscillation activities for stronger coupling. From what we have mentioned above, we simulate four different dynamical states of the cerebral cortex via the slow kinetics of GABA_B_ receptors and coupling strength −*v*_*sr*_ of TRN-SRN pathway. In particular, the typical absence epilepsy state can be successful replicated. Throughout the whole process, we set τ = 50*ms*, which pertains to the physiological range, contributing to the generation of 2–4 Hz SWD oscillation patterns.

### 3.2. Control of absence seizures by the DBS to TRN

DBS of the thalamus is a new and developing therapy, which is effective for the refractory epilepsy patients who are not suitable for pharmacotherapy or resective surgery (Valentín et al., 2013). Previous researchers utilized many animal and clinical experiments to confirm the stimulating efficacy for different brain structures, such as the subthalamic nucleus (Chabardès et al., 2002), the centromedian thalamic nucleus (Valentín et al., 2013), Anterior nucleus of the thalamus (Mirski et al., 1997), thalamic reticular nucleus (Nanobashvili et al., 2003) and the cortical regions (Morrell, 2011). The thalamus as a crucial nervous center is closely related to epileptic seizures. Here, we study the effect of the DBS to TRN on the generation and elimination of absence seizures. As shown in Figure 1, the TRN neurons send the inhibitory signals mediated by both GABA_A_ and GABA_B_ receptors to SRN. After the firing of SRN is inhibited by the GABA_A_ receptors, the SRN neurons need some time to recover to their initial state. Meanwhile, the GABA_B_-induced inhibition will generate the delay τ. Therefore if the delay τ is longer than the recovery time, the SRN neurons can induce another firing peak. Hence the relatively large delay τ is easy to produce absence epilepsy activities. For a stronger −*v*_*sr*_, the GABA_A_-induced inhibition is also strong. In this situation, the SRN neurons need a longer time to restore their firing rate. Therefore, a relatively long τ is required to ensure the occurrence of SWDs for stronger −*v*_*sr*_. In addition, the DBS mainly affects the interactions among different neuron populations in our model, i.e., the range of delay τ in pathological region remains nearly unchanged. Based on above discussions, if we want to control absence seizures, the most straightforward method is to stimulate TRN because the TRN stimulation contributes to a stronger inhibitory effect.

**Figure 4:**
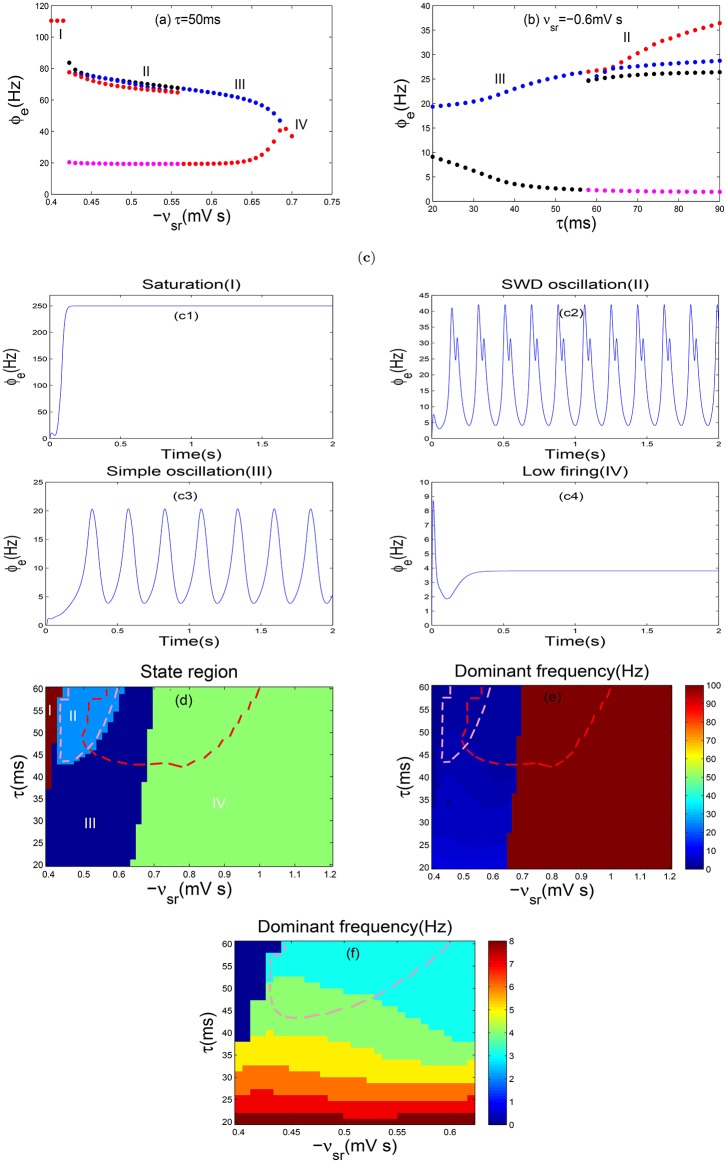
**Controlling absence seizures through the DBS to TRN**. **(a,b)** The bifurcation diagrams of ϕ_*e*_ as a function of −*v*_*sr*_ **(a)** and τ **(b)**. In order to observe the bifurcation phenomenon obviously, the ordinate scale is used exactly in a log scale in **(a)**. **(c)** The typical time series of ϕ_*e*_ are corresponding to the above four different dynamics states. Here we choose −*v*_*sr*_ = −0.4 mV s **(c1)**, −*v*_*sr*_ = −0.5 mV s **(c2)**, −*v*_*sr*_ = −0.6 mV s **(c3)**, −*v*_*sr*_ = −0.7 mV s **(c4)** and set τ = 50 ms. **(d–f)** Two-dimensional state analysis **(d)** and the frequency analysis **(e)** in the (−*v*_*sr*_, τ) panel. The enlargement **(f)** here is a local focus on **(e)**, with the purpose of observing a obvious phenomenon. Different colors in **(d)** represent the different dynamical states: the saturation state (I), the SWD oscillation state (II), the simple oscillation region (III), the low firing state (IV). The region surrounded by pink dashed lines in **(d,e)** represents the SWDs falling into the 2–4 Hz frequency range after adding the DBS to TRN, however the regions surrounded by red dashed lines stand for the 2–4 Hz SWDs without DBS. The SWD oscillation pattern has a obvious reduction, i.e., the absence seizures can be eliminated through the DBS to TRN.

Similarly, one-dimensional bifurcation diagrams are plotted with regard to the inhibitory coupling strength −*v*_*sr*_ (Figure 4a) and the delay parameter τ (Figure 4b), respectively. As shown in Figure 4a, we find four different firing states as the parameter −*v*_*sr*_ is changed, and their corresponding time series are depicted in Figures 4c1–c4. It is clear that the transitions in Figures 4a,b are similar to those in Figures 3a,b without DBS. However, further investigations find that the range of parameters −*v*_*sr*_ and τ for different dynamical states are changed. Especially, the absence epilepsy state within the range of parameters −*v*_*sr*_ and τ is reduced significantly. For more details, we perform the state analysis in the the two-parameter plane (−*v*_*sr*_, τ). As shown in Figure 4d, the region surrounded by red dashed lines represents the SWD oscillation region (II) in Figure 3d, and the region surrounded by pink dashed lines is the SWD oscillation region as the DBS is applied. Comparing the two regions of Figures 3d, 4d, we find that the region (II) has an obvious reduction in Figure 4d. Additionally, the dominant frequency is shown in Figure 4e to estimate the frequency characteristics of different oscillation patterns. For clearer vision, in Figure 4f, we enlarge the SWD oscillation region of Figure 4e, which indeed falls into 2–4 Hz frequency range. The stimulation parameters we used keep within a reasonable range, which have been applied in previous studies (Mirski et al., 1997; Usui et al., 2005). Therefore, we can conclude that the DBS can eliminate absence seizures.

### 3.3. Effects of DBS on SWD oscillation pattern with different parameters

So far, we have confirmed that absence seizures can be simulated and eliminated in our simplified mean-field model. The choice of the stimulation parameters including frequency, amplitude and positive input duration is also critical to the theoretical and clinical researches. Therefore, we continue to investigate the influence of different stimulation parameters on the SWD oscillation region in the following studies.

**Figure 5:**
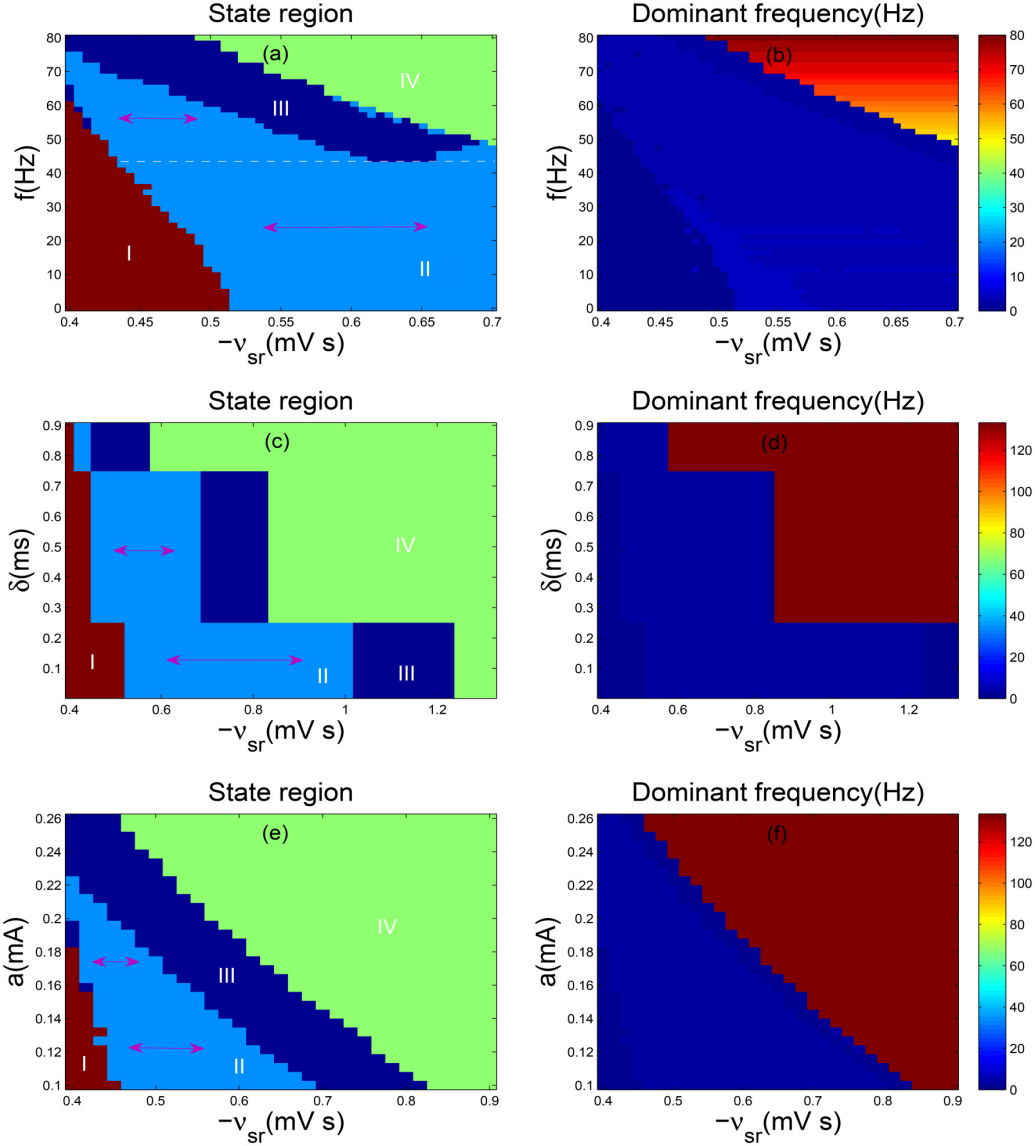
**(a,b)** Two-dimensional state analysis **(a)** and corresponding dominant frequency analysis **(b)** in the (−*v*_*sr*_, *f*) panel; **(c,d)** The state analysis **(c)** and frequency analysis **(d)** in the (−*v*_*sr*_, δ) panel; **(e,f)** The state analysis **(e)** and frequency analysis **(f)** in the (−*v*_*sr*_, *a*) panel. Here we set δ = 2 ms, *a* = 0.1 mA **(a,b)**; *f* = 130 Hz, *a* = 0.1 mA **(c,d)**; *f* = 130 Hz and δ = 0.7 ms **(e,f)**. −*v*_*sr*_ represents the inhibitory coupling strength between TRN and SRN, whereas *f* denotes frequency, δ is positive input duration and *a* is stimulation amplitude. Different colors in **(a,c,d)** represent different dynamics states: the saturation state (I), the SWD oscillation state (II), the simple oscillation state (III), the low firing state (IV). Double sided arrow represents parameter interval of TRN-SRN inhibitory coupling strength −*v*_*sr*_ in the SWD oscillation region, and white dashed line stands for the largest one in **(a)**. For all simulations, we set delay τ = 50 ms.

Currently, we will explore the control effect of DBS current on absence seizures when we change the frequency *f*. As shown in Figure 5a, two-dimensional state analysis has been obtained and the white dashed line represents the largest parameter interval of the inhibitory coupling strength −*v*_*sr*_ in the SWD oscillation region. For the section below the white dotted line, when the frequency *f* is decreased, the parameter interval of −*v*_*sr*_ in the SWD oscillation region diminishes slowly, i.e., the suppression of SWDs can be achieved slowly. Whereas for the section above the white dotted line, the parameter range of −*v*_*sr*_ in the SWD oscillation region reaches the minimum value quickly as the *f* is increased, i.e., the SWDs can be inhibited by raising the parameter *f*. Here the double arrow represents the parameter interval of −*v*_*sr*_ in the SWD oscillation region. Moreover, the corresponding dominant frequency of Figure 5a is shown in Figure 5b. Although the distribution of frequency seems unclear, the SWD oscillation state indeed falls into 2–4 Hz frequency range. On the whole, DBS strongly reduce the persistent absence seizures for low-frequency (<10 Hz) and high-frequency (>60 Hz) while intermediate frequency stimulation (around 45 Hz) have no effect. These results are nearly similar with previous researches (Rubin and Terman, 2004; Mina et al., 2013).

We further discuss the stimulation parameter δ. As presented in Figure 5c, the bifurcation diagram looks very regular, especially, the SWD oscillation region is divided into three parts, each one is similar to a rectangle. For a relative weaker δ, the parameter range of −*v*_*sr*_ in the SWD oscillation region achieves the maximum value and keeps unchanged. With increasing δ, the interval of −*v*_*sr*_ in the SWD oscillation region obviously decreases. Especially, when the positive input duration δ exceeds about 0.75*ms*, the interval of SWDs almost disappears. It is clear that the SWDs can be eliminated through increasing the parameter δ. Figure 5d illustrates the corresponding dominant frequency analysis of Figure 5c and the corresponding SWD oscillation state falls into 2–4 Hz frequency range. At last, we investigate the amplitude *a*. The two-dimensional bifurcation diagram and frequency characteristic are demonstrated in the plane (−*v*_*sr*_, *a*). As shown in Figure 5e, as *a* is increased, the parameter interval of the SWD oscillation region about inhibitory coupling strength −*v*_*sr*_ becomes smaller before it disappears. As shown in Figure 5f, the typical absence seizures falls into 2–4 Hz frequency range. Hence, it is shown that the suitable stimulations can contribute to the elimination of absence seizures through the DBS to TRN.

## 4. Conclusion

Taking advantage of the mean-field macroscopic model which associates with the thalamus and the cerebral cortex, we have studied how the deep brain stimulation eliminates the absence seizures. The obtain results have shown that the appearance of some transition states, definitely including absence seizures in our model, results from the change of the coupling strength of TRN-SRN pathway and the slow synaptic kinetics of GABA_B_ receptors on TRN. We have also found that a relatively long delay τ is required to assure the occurrence of SWDs for stronger −*v*_*sr*_. Combining with some existing animal experiments, we choose the TRN as a suitable stimulation target in this paper. The detail dynamics and qualitative analysis have shown that adding the suitable DBS to TRN contributes to an obvious reduction of the SWD oscillation region. We have further investigated how the corresponding stimulation parameters including frequency, amplitude and positive input duration suppress the SWDs under certain conditions. The results have indicated that suitable stimulations can eliminate absence seizures as the DBS is applied to TRN. However, it is well known that a human's brain is quite different from a rodent's and clinical trials can't be replaced by animal models. Thus, the information from animal models only can guide clinician to explore how the treatment is applied to human. Hence, we hope that our computational results can provide a theoretical guidance for the treatment of real absence epilepsy patients.

## Author contributions

ZW and QW designed and performed the research as well as wrote the paper.

### Conflict of interest statement

The authors declare that the research was conducted in the absence of any commercial or financial relationships that could be construed as a potential conflict of interest.
